# Activated Mesenchymal Stromal Cell Therapy for Treatment of Multi-Drug Resistant Bacterial Infections in Dogs

**DOI:** 10.3389/fvets.2022.925701

**Published:** 2022-06-23

**Authors:** Valerie Johnson, Lyndah Chow, Jacqueline Harrison, Sirikul Soontararak, Steven Dow

**Affiliations:** ^1^Department of Clinical Sciences, Center for Immune and Regenerative Medicine, College of Veterinary Medicine and Biomedical Sciences, Colorado State University, Ft. Collins, CO, United States; ^2^Department of Small Animal Clinical Sciences, College of Vetinerary Medicine, Michigan State Univeristy, East Lansing, MI, United States; ^3^Department of Companion Animal Clinical Sciences, Faculty of Veterinary Medicine, Kasetsart University, Bangkok, Thailand

**Keywords:** antibiotic, mesenchymal stem cells, bacteria, drug resistance, immune, antimicrobial

## Abstract

New and creative approaches are required to treat chronic infections caused by increasingly drug-resistant strains of bacteria. One strategy is the use of cellular therapy employing mesenchymal stromal cells (MSC) to kill bacteria directly and to also activate effective host immunity to infection. We demonstrated previously that activated MSC delivered systemically could be used effectively together with antibiotic therapy to clear chronic biofilm infections in rodent models. Therefore, we sought in the current studies to gain new insights into the antimicrobial properties of activated canine MSC and to evaluate their effectiveness as a novel cellular therapy for treatment of naturally-occurring drug resistant infections in dogs. These studies revealed that canine MSC produce and secrete antimicrobial peptides that synergize with most classes of common antibiotics to trigger rapid bactericidal activity. In addition, activated canine MSC migrated more efficiently to inflammatory stimuli, and secreted factors associated with wound healing and fibroblast proliferation and recruitment of activated neutrophils. Macrophages incubated with conditioned medium from activated MSC developed significantly enhanced bactericidal activity. Clinical studies in dogs with chronic multidrug resistant infections treated by repeated i.v. delivery of activated, allogeneic MSC demonstrated significant clinical benefit, including infection clearance and healing of infected tissues. Taken together, the results of these studies provide new insights into antimicrobial activity of canine MSC, and their potential clinical utility for management of chronic, drug-resistant infections.

## Introduction

The unrelenting increase in bacterial resistance continues to stymie medical advances in antimicrobial therapy, and pose new challenges for the medical community to develop creative solutions, which necessarily will involve more than just new antibiotics (1–3). Drug-resistant infections are also increasingly common in veterinary medicine, as noted in recent reviews (4, 5). Therefore, there is an urgent need for creative new strategies for management of drug-resistant infections in both human and animal health.

Cellular therapy with mesenchymal stromal cells (MSC) is a promising new strategy to directly and indirectly target bacteria for killing and elimination, and the creative use of MSC as host derived antimicrobial effectors has been described by our group and others (6–17). Mechanisms of bacterial clearance include direct bactericidal activity of secreted antimicrobial peptides, as well as engagement and activation of host innate anti-bacterial defenses. Examples of successful use of MSC to treat acute infections have been described in a number of rodent models of acute bacterial pneumonia and sepsis (10, 11, 15, 18, 19). Clinical trials of MSC therapy for treatment of bacterial pneumonia and ARDS (Acute respiratory distress syndrome) are currently underway (14).

However, relatively less investigation has been done with MSC for the treatment of chronic, drug-resistant infections such as MRSA (Methicillin-resistant *Staphylococcus aureus* infections). Chronic infections are often associated with biofilm formation, rendering the infection much more refractory to long-term antibiotic therapy. However, we have recently shown that TLR-activated MSC can be used effectively to clear *S. aureus* in a rodent model of chronic biofilm infection, in combination with antibiotic therapy (10). In addition, we and others have shown that MSC secreted antimicrobial peptides can delay and disrupt biofilm formation *in vitro* (7, 9). Thus, the use of MSC therapeutically for management of intractable, drug-resistant infections in difficult to reach tissues such as bone and deep fascia is an attractive alternative to antibiotic only therapy.

In the current study, we extended our prior observations with human and murine MSC to now include canine MSC and their antimicrobial properties, in part because of the growing importance of drug-resistant infections in veterinary medicine (4). We investigated potential mechanisms, both direct and indirect, to explain canine MSC bactericidal activity, and interactions with common classes of antibiotics. The effectiveness of activated canine MSC as a new tool for clinical management of drug-resistant infections was investigated in a trial of i.v. administered, activated canine MSC in pet dogs with naturally-occurring drug-resistant infections. These investigations determined that antimicrobial therapy with activated allogeneic MSC in dogs was well-tolerated, was associated with rapid infection clearance and healing, and suggested that additional investigation of this novel anti-infective strategy was warranted.

## Materials and Methods

### Generation of Canine Adipose Derived MSC

Adipose tissues were obtained from the inguinal region of healthy, young purpose bred research hounds used in unrelated teaching laboratory studies, under a CSU Institutional Animal Care and Use Committee approved protocol. The adipose tissue was frozen in 1 gm aliquots in freezing media consisting of 75% FBS, 15% tissue culture medium, and 10% DMSO, using slow rate freezers to a final temperature of −80 C, within 2 h of sample collection. Fresh cultures of MSC were established following thawing of the tissues and collagenase digestion, as described previously (20). Briefly, the adipose tissue was minced, digested in collagenase, the stromal vascular fraction collected, and the adherent MSC expanded in tissue culture flasks as previously described. (10). The MSC were also characterized by flow cytometry (data not shown) for characteristic phenotype and for expression of MSC-associated markers (CD90, CD73, CD105) and for markers associated with contaminating hematopoietic cells (CD34, MHCII), using protocols described previously (20). Cells for both *in vitro* and *in vivo* studies were used between passages 2 and 5.

Cells were cultured in complete DMEM (Thermo Fisher Scientific, Grand Island, NY) containing 10% FBS (VWR-Seradigm, Inc., Aurora, CO), 2 mM l-glutamine, 100 U/mL penicillin G, and 100 ug/mL streptomycin (Thermo Fisher Scientific) as described previously (20).

### MSC Activation by TLR3 Agonist

To activate MSC to enhance their antimicrobial activity, as reported previously (10), the cells were incubated with high molecular weight poly-inosinic, poly-cytidylic acid (pIC) (InvivoGen, San Diego, CA), at a concentration of 10 ug/mL for 2 h at 37°C. Following the activation step, the pIC was removed by washing with PBS, and the MSC were detached from flasks by incubation with trypsin-EDTA solution (Thermo Fisher Scientific) prior to use in assays or for *in vivo* treatment.

### Generation of Conditioned Media for *in vitro* Studies

For use in *in vitro* studies, MSCs were cultivated from adipose using complete media without the addition of penicillin G and streptomycin starting from the first passage. Activated MSCs were generated as described above (activated with pIC for 2 h at 37°C, washed twice with PBS). Conditioned medium was generated by plating 5 × 10^5^ cells per well (passage 3–5) in a 24 well-plate with 500 ul per well of antibiotic free media and incubating at 37 °C. Conditioned media was collected 24 h after the cells were plated and were immediately frozen at −80 °C and thawed immediately before use.

### Skin Fibroblast Cultures

Canine skin fibroblasts were generated from skin samples from three different healthy research bred dogs (see above) as described previously (21). Skin biopsy was taken using a 6 mm skin biopsy punch (Miltex, York, PA). and placed in a 6 well cell culture dish (Corning Inc. Corning, NY) and cultured in complete DMEM. Fibroblasts outgrowths were collected and used for *in vitro* assays.

### Edu Incorporation for Proliferation

Skin fibroblasts were plated at a concentration of 50,000/mL in a 24 well cell culture plate (Corning Inc. Corning, NY) and allowed to adhere for 4 h. Conditioned media was added to fibroblasts and incubated for 48 h; fibroblasts were then detached using trypsin-EDTA solution (Thermo Fisher Scientific). Proliferation was measured at 48 h using Click-iT® Plus EdU Alexa Fluor® 647 Flow Cytometry Assay Kit (Thermo Fisher Scientific) following manufacturer protocols, with 10 uM of EdU (5-ethynyl-2 -deoxyuridine) addition 24 h prior to assay conclusion. Cells were analyzed on a Beckman Coulter Gallios flow cytometer (Brea, CA), and the flow cytometry data were then were analyzed using FlowJo Software v10.5 (Ashland, OR).

### Monocyte-Derived Macrophage Cultures

Canine monocyte-derived macrophages were generated by growth in 10 ng/mL of human M-CSF (Macrophage colony-stimulating factor) (Peprotech, Rocky Hill, NJ), as previously described (22). Briefly, monocytes were collected from the blood of unrelated, healthy dogs, using Ficoll-Paque PLUS (Sigma Sigma-Aldrich, St. Louis, MO) separation gradients according to manufacturer's instructions. Monocytes were separated by adherence to plastic cell culture dishes overnight, with non-adherent cells washed off, and the remaining adherent monocytes were then differentiated to macrophages following 7 days in M-CSF (macrophage colony stimulating factor) (PeproTech, Cranbury, NJ) supplemented complete medium. Media and M-CSF were replaced every 3 days, removing non-adherent cells each time.

### Macrophage Immunocytochemistry

Monocyte-derived macrophages were cultured in chamber cover glass system (Cellvis, Mountain View, CA) for up to 7 days then fixed using 4% paraformaldehyde (Fisher Scientific, Hampton NH). Macrophages were then permeabilized using 0.1% Triton X (Thermo Fisher Scientific), and blocked with normal donkey serum (Jackson ImmunoResearch Laboratories Inc. West Grove, PA). Primary antibodies (or irrelevant isotype matched antibodies) CD206-PE (macrophage mannose receptor) (Clone 3.29B1.10) (Beckman Coulter, Brea CA), rabbit polyclonal anti-mouse iNOS, Inducible nitric oxide synthase (# PA3-030A, Thermo Fisher Scientific), were then added and incubated overnight at 4°C. Negative controls included cells that were immunostained with isotype-matched, irrelevant target antibodies, including mouse IgG1 (Thermo Fisher) or ChromPure Rabbit IgG (Jackson ImmunoResearch Laboratories Inc). Following overnight incubation with primary antibodies, cells were stained with appropriate secondary antibodies AffiniPure donkey anti-rabbit or anti-mouse IgG (Jackson ImmunoResearch Laboratories Inc) for 1 h then counter stained with DAPI (diamidino-2-phenylindole) for nuclear staining (Thermo Fisher Scientific). Slides were examined under fluorescence microscopy using an Olympus IX83 spinning disk confocal microscope (Olympus Scientific Solutions Americas & Olympus America Inc. Waltham, MA). A series of 10 random fields per slide were imaged at 20X magnification, and the images were than subject to image quantification, using ImageJ software https://imagej.nih.gov/ij/, and the median fluorescence intensity (MFI) for marker expression was calculated. M2/M1 ratio was calculated by dividing MFI of CD206 (M2 marker) by iNOS (M1 marker).

### Cell Migration Assays for MSC and Fibroblasts

Migration of MSC toward an inflammatory stimulus (SDF-1, stromal cell-derived factor 1 or CXCL12) was assessed utilizing transwell migration chambers with an 8 um pore diameter (BD Falcon). Cell migration was stimulated by 100 ng/ml SDF-1 (R&D Systems Inc., Minneapolis, MN) or MSC conditioned medium placed in the bottom chambers of triplicate wells of the transwell system. The MSC were allowed to migrate for 4 h while incubating at 37°C 5% CO_2_, then the chamber inserts were removed and migrated cells in the bottom chamber were fixed in 2% methanol and stained with crystal violet. The migrated cells were counted manually and an average of 10 random fields at 10X magnification were photographed using an Olympus IX83 spinning disk confocal microscope, and the average of all 10 fields determined for each well.

Fibroblast migration was assessed using an Incucyte instrument (Sartorius AG, Göttingen, Germany), and a scratch assay, according to manufacture protocol. Briefly, 1 X 10^4^ skin fibroblasts per well were plated in a triplicate wells of 96-well-culture plate and allowed to attach overnight. Uniform scratches were then made in the center of each well of fibroblasts using an Incucyte tool. The area of cell coverage at the margins of the scratch defect was measured for each well at 12, 24 and 48 h.

### Measurement of MSC Secreted Cytokines

Conditioned media from activated and resting MSC was collected after 48 h in culture, as described above and frozen at −80°C until use. Cytokine concentrations were analyzed using a commercial canine IL-8 ELISA kit (R&D Minneapolis MN), according to manufacturer directions.

### Fibroblast Viability Assay

Fibroblast viability was assessed using an MTT assay, which utilized (3-(4,5-dimethylthiazol-2-yl)-2,5-diphenyl tetrazolium bromide; MTT) (Sigma-Aldrich). The MTT solution was added to 24-well-plates with triplicate cultures, at a 5 ug MTT solution per ml medium concentration, followed by incubation for 2 h at 37°C. To stop the MTT reaction, 250 ul of 0.01 M HCL was added to the wells to solubilize the formazan crystals and the resulting colored solution was quantitated using an optical density reader.

### Antimicrobial Peptide Expression by MSC Using Immunocytochemistry

Immunocytochemical staining of MSC for expression of antimicrobial peptides was performed as described previously (9, 16). Briefly, 1 X 10^4^ cells were seeded on round coverslips (Chemglass Life Sciences, Vineland, NJ) placed within 24-well-cell culture plates overnight, then fixed with 4% paraformaldehyde (Fisher Scientific, Hampton, New Hampshire) for 10 min, washed with PBS and permeabilized with 0.1% Triton X-100(Sigma-Aldrich,). Slides were then blocked using 5% v/v normal donkey serum and then incubated with primary antibodies, diluted appropriately. Antibodies used for these studies included surfactant protein D antibody (ab203309), lipocalin-2 antibody (ab63929), beta 2 defensin antibody (ab9871), hepcidin antibody (ab134790), and cathelicidin antibody (ab180760), all obtained from Abcam (Cambridge, MA). Specificity controls for immunostaining included purified IgG antibodies from non-immune rabbits or goats. Following primary antibody incubation, slides were washed and incubated with secondary antibodies, either donkey anti-rabbit or donkey anti-goat, both conjugated to Cy3 (Jackson ImmunoResearch Laboratories, Inc, West Grove, Pennsylvania) and the slides were then counter-stained with DAPI to visualize cell nuclei. Image capture for fluorescence staining was done using an Olympus IX83 spinning disk confocal microscope.

### Macrophage Bacterial Phagocytosis Assay

Bacterial phagocytosis by macrophages was assessed using an Incucyte assay system (Essen BioScience Inc, Ann Arbor, Michigan). To conduct the assay, log phase *S. aureus* cultures were first fixed and stained using the pH rodo Red Phagocytosis Particle Labeling Kit (Thermo Fisher Scientific), according to manufacturer's instructions. The impact of MSC conditioned medium on macrophage phagocytosis was assessed by incubating triplicate cultures of canine monocyte-derived macrophages with MSC conditioned medium for 48 h. Next, macrophages were inoculated with *S. aureus* at an MOI (multiplicity of infection) of 25:1 (bacteria to cells) for 1–3 h. Images from infected cells in the Incucyte system were collected every 15 min using a 10X objective and analyzed using IncuCyte S3 Software (Essen BioScience Inc).

### Macrophage Bactericidal Assay

Monocyte-derived macrophages, generated as noted above, were infected with drug-resistant *S. aureus*, at a MO1 of 3:1 (3 CFU/cell), diluted in HBSS containing Ca^++^ and Mg^++^ and 10% dog serum, and incubated for 1 or 3 h, as described previously (23, 24). After 1 h incubation with bacteria, the non-engulfed bacteria were washed extensively from the adherent macrophages, baseline bacterial count was taken first by lysing macrophages briefly in distilled water to free intracellular bacteria. After an additional 2 h of incubation (T180), additional wells of macrophages (with and without MSC CM or aMSC CM) were lysed to compare to baseline. These macrophage lysates were then plated at log 10 serial dilutions on LB agar 4 quadrant plates (Thermo Fisher Scientific) and colony forming units (CFU) determined after 24 h of incubation at 37°C. Percent bacterial killing as a relative percentage decrease in CFU/mL of T180 compared to basline bacterial counts.

### Bacterial Strains and Propagation

Bacterial isolates utilized in *in vitro* studies were obtained courtesy of the Colorado State University Veterinary Diagnostic Laboratories. Isolates of *Escherichia coli* (*E Coli*) and *Staphylococcus pseudointermedius* (*S. pseudointermedius*) were obtained from wound cultures received by the bacteriology laboratory from hospitalized canine patients with informed consent. A single bacterial colony from the plate was used to inoculate trypticase soy broth (Sigma-Aldrich) at 37°C in a shaking incubator until the culture was turbid (~8 h). Sterile glycerol (Sigma-Aldrich) was added at a concentration of 20% and aliquots of 1 ml were frozen at −80°C until use. *S. pseudointermedius* was identified by growth on 5% Sheep blood agar (BD Biosciences, Franklin Lakes, NJ) with double zone hemolysis which was coagulase positive and hyaluronidase negative with gram positive cocci identified on gram stain. *E coli* was identified by growth on MacConkey agar (BD Biosciences) that was a strong lactose fermenter and was indole negative with the presence of gram negative rods on gram stain. Antimicrobial susceptibilities were performed utilizing the Kirby Bauer method in accordance with the Clinical Laboratory Standards Institutes veterinary guidelines (CLSI vet) (25).

### Bacterial Quantitative Culture

Bacterial numbers were quantitated by plating serial, log 10 dilutions of culture supernatants obtained by gentle pipetting, and then plating on LB agar quadrant plates (Sigma-Aldrich) and counting bacterial colonies 24 h after culture at 37 °C. The log 10 CFU was determined numerically from serially diluted plate counts.

### Bactericidal Assays for Assessment of MSC Secreted Antimicrobial Factors and Interactions With Antibiotics

The ability of MSC secreted factors to induce bacterial killing, cells or conditioned medium (CM) from MSC were utilized. Conditioned medium was generated by plating 5 × 10^5^ cells per well in a 24 well-plate with 500 ul per well of antibiotic free media and incubating at 37 °C. Conditioned media was collected 24 h after the cells were plated and were immediately frozen at −80 °C and thawed immediately before use. Either live MSC, or MSC CM, was inoculated with log phase *Staphylococcus aureus* cultures, at a concentration of 1 × 10^5^ CFU bacteria per ml of CM or per ml of cells (multiplicity of infection of 10:1 bacteria per cell) in 24-well-plates, in complete MSC culture medium, without antibiotics, as described previously (23). Cultures were incubated at 37 °C for 3h, then supernatants removed and numbers of viable bacteria determined, as noted above. Antibiotics used to investigate interactions with MSC secreted factors were used at sub-therapeutic concentrations to elucidate the effects on antibiotic activity.

### Bacterial Quantitative Cultures From Wound Samples

The bacterial burden in wound tissues of dogs in the clinical trial were determined by collecting 4-quadrant fine needle aspirates from the wound perimeter, The aspirates were placed in 1 ml of TSB (trypticase soy broth, Sigma Aldrich) which were then each inoculated into LB medium then serially diluted and plated on LB plates and incubated for 24 h for quantification. The inoculated broth was also plated on sheep blood agar (TSA with sheep blood) plates and MacConkey plates (BD Falcon, San Diego CA) for bacterial identification. Plates were incubated for a minimum of 48 h at 37 °C before concluding negative bacterial growth. For animals with septic arthritis, a 0.25–0.5 ml synovial fluid sample was aspirated and cultured at each of the 2-week sampling intervals, and the synovial fluid was also evaluated cytologically. Animals with nasal infections underwent periodic surveillance of bacterial populations in the affected nares by culturing nasal swab samples. Bacteria were speciated by morphology and biochemical testing, and bacterial CFU were determined by manual counting. Bacterial sensitivities were performed by the Colorado State Veterinary Diagnostic Laboratory.

### Clinical Trial Design for Dogs With Drug-Resistant Chronic Infections

Dogs with spontaneously occurring, drug-resistant infections evaluated at the CSU Veterinary Teaching Hospital (VTH) for treatment were recruited for enrollment into a clinical trial evaluating the safety and efficacy of activated allogeneic canine MSC therapy, in a study design that was described previously (10). A total of 8 dogs were enrolled in this study. These studies were approved by the Colorado State University Institutional Animal Care and Use Committee and the Clinical Review Board at the Veterinary Teaching Hospital. Inclusion criteria included documented infection with multi-drug resistant bacteria (single or multiple species), and an infection that had failed to respond to conventional antibiotic treatment for a period of at least 4 weeks. Animals were excluded from study if they had concurrent diseases associated with endogenous immune suppression, including cancer, Cushing's disease, kidney disease, or diabetes mellitus, or if they were being treated concurrently with immune suppressive drugs (eg, corticosteroids or oclacitinib).

The study was designed to last 8 weeks, during which study animals received 3 infusions of activated canine MSC at 2-week intervals, with 2 weeks for follow-up after the last cell infusion. The MSC dose for each dog was 2 × 10^6^ cells per kg body weight, and MSC were activated with pIC as noted in Methods for 2 h prior to infusion. After activation, MSC were detached and counted, and resuspended in PBS, phosphate buffered saline pH 7.4 (typically 10–20 ml final volume) for i.v. infusion. Cells were administered through a peripheral vein catheter over 15 min with continuous monitoring of heart rate and respiratory rate. Importantly, for all study animals, the original ineffective antibiotic therapy was kept unchanged for the duration of the 8-study, to eliminate antibiotic selection as a variable. Dogs were evaluated clinically at each study visit, and infection site cultures for quantitative culture whenever possible. The study design also allowed for animals that completed the 8-week trial, without complete infection resolution, to continue to receive additional activated MSC treatments, at the discretion of the study trial director and attending clinicians.

### Assessment of Bacteriologic and Clinical Responses

Study dogs were evaluated every 2 weeks for 8 weeks to assess clinical and bacteriological responses. Criteria for a microbiological response included a decrease in 50% or more of quantitative bacterial counts, compared to pre-treatment values. In animals where quantitative cultures were not possible, an animal was considered to be a responder if previously positive cultures reverted to negative during the study period. Partial responders were defined as dogs with elimination of some, but not all of the bacterial species, in cases of polymicrobial infections.

A clinical infection response was defined depending on the type and site of infection. For dogs with open wounds, the wounds were measured at each two-week timepoint and a clinical response defined as >50% contraction of the wound. For dogs with joint infections, a clinical response was defined as reduction of inflammation in the joint as assessed by numbers of inflammatory cells in joint fluid (>50% decrease in cell count in synovial fluid). In the case of two dogs with nasal infections, responses were assessed by owners who scored nasal discharge and sneezing with a numeric score, ranging from 0 (no signs) to 4 (severe signs).

### Statistical Analyses

Statistical analysis was performed using Graph Pad Prism 8.0 software (Graph Pad, La Jolla CA). For comparison of two treatment groups a *t*-test was performed (Mann-Whitney test). For comparison of more than two groups a two-way ANOVA was run with a Tukey post-test. Tests for synergy were performed using a two-way ANOVA as previously described (26).

## Results

### Canine MSC Exhibit Synergistic Bactericidal Activity When Combined With Antibiotics *in vitro*

We first sought to identify classes of antibiotics that might exhibit synergistic or additive activity with bactericidal factors secreted by resting or activated MSC. These studies were also done to guide antibiotic selection for combined treatment with MSC clinically. The screens were performed using *in vitro* bactericidal assays, using subtherapeutic drug concentrations to elucidate the interaction of antibiotics with MSC secreted factors. Two common bacterial isolates from dogs with chronic infections were used in these studies (antibiotic resistant *Staphylococcus pseudointermedius* and antibiotic-resistant *Escherichia coli*), in part because we wished to determine whether Gram-positive and Gram-negative bacteria interact differently with MSC secreted factors and antibiotics. A subinhibitory concentration for each antibiotic was identified by determining the MIC (minimum inhibitory concentration) for each bacterium and antibiotic, and then reducing the drug concentration used in the assays 10-fold.

**Figure 1:**
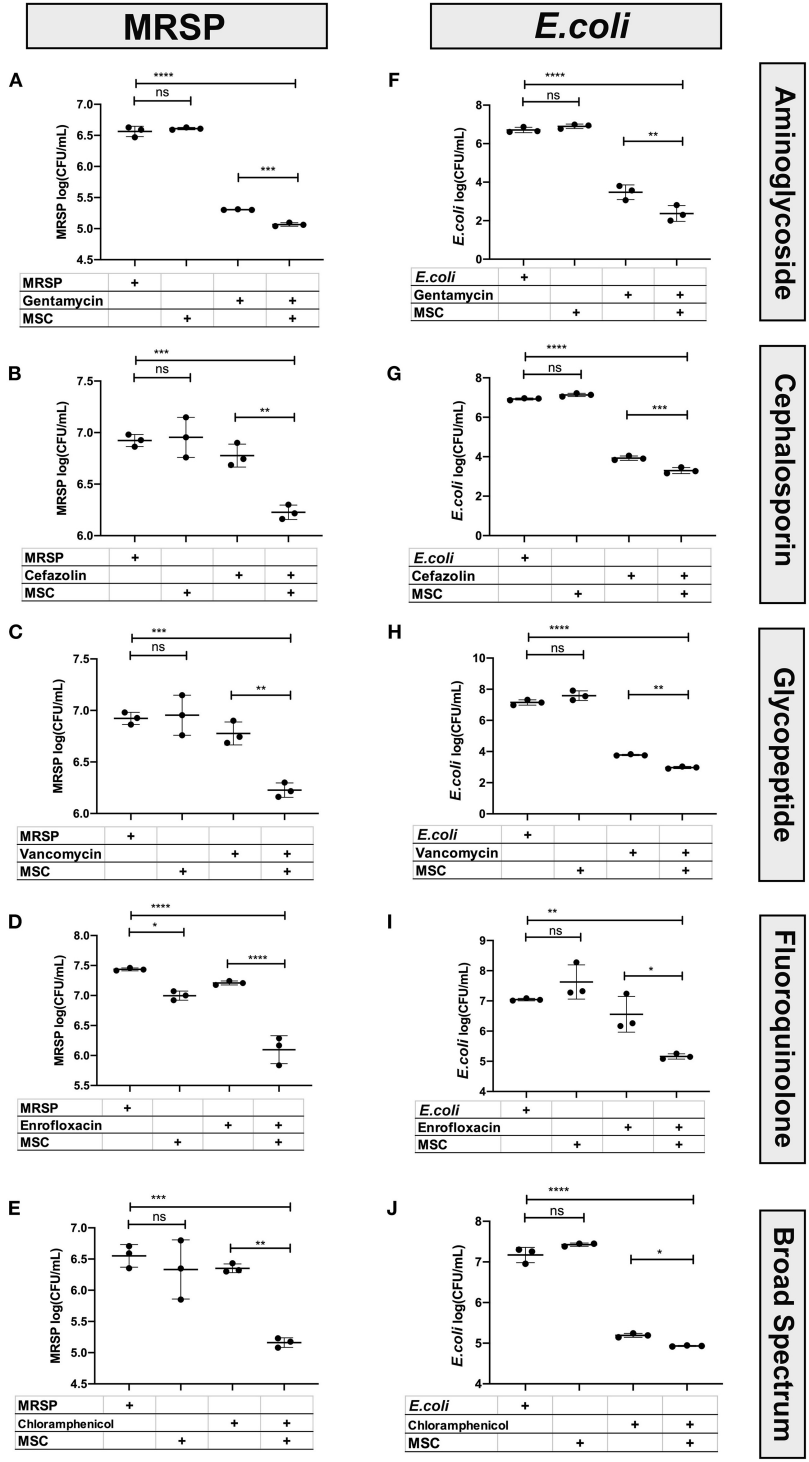
Interaction of antibiotics and MSC secreted factors for induction of bactericidal activity. Bactericidal assays were used to evaluate the interaction between MSC and clinically relevant antimicrobials used in the management of chronic drug-resistant infections in dogs, as described in Methods. The left column **(A–E)** depicts bactericidal assays performed with S. pseudointermedius, while the right-hand column **(F–J)** depicts results from E coli. The y-axis depicts the log10 bacterial CFU/mL. Synergy was computed using an interaction factor and Prism 8.0 software (GraphPad, San Diego, CA) two-way ANOVA as described previously (10, 26). Comparisons between three or more groups were done using one-way ANOVA, followed by Tukey multiple means post-test. Statistical significance was determined for *P ≤ 0.05, **P ≤ 0.01, ***P ≤ 0.001, ****P ≤ 0.0001.

These studies revealed that MSC and their secreted factors in conditioned medium (CM) exhibited additive or synergistic bactericidal activity when co-incubated with all of the major classes of antibiotics tested, and with both *Staphylococcus* and *E coli* (Figure 1). For example, while MSC CM alone did not exhibit significant direct bactericidal activity, when the MSC CM was combined with subtherapeutic antibiotic concentrations, there was a significant and often synergistic bactericidal activity. Bactericidal synergy was demonstrated for aminoglycosides (gentamicin) (Figures 1A,F), cephalosporins (cefazolin) (Figures 1B,G), vancomycin (Figures 1C,H), fluoroquinolones (enrofloxacin) (Figures 1D,I), and chloramphenicol (Figures 1E,J). Taken together, these findings indicate that cellular therapy with activated MSC can be effectively used with all of the major classes of antibiotics evaluated, including bacteriostatic drugs such as chloramphenicol.

### Canine MSC Constitutively Express Antimicrobial Peptides

Previous studies in human, rodent, and also equine models have shown that MSC express a variety of antimicrobial peptides important in direct bacterial killing, including LL-37 (cathelicidin), hepcidin, lipocalin, β-defensin, and surfactant protein D (6, 7, 9, 12, 15). Each of these antimicrobial peptides play important roles in MSC-mediated killing of both Gram negative and Gram-positive bacteria, typically by creating pores in bacterial membranes. In addition, previous studies have established that several of these antimicrobial peptides display synergistic bactericidal activity when combined with antibiotics, which would be predicted based on the peptide mechanism of action (6, 10, 12, 13).

**Figure 2:**
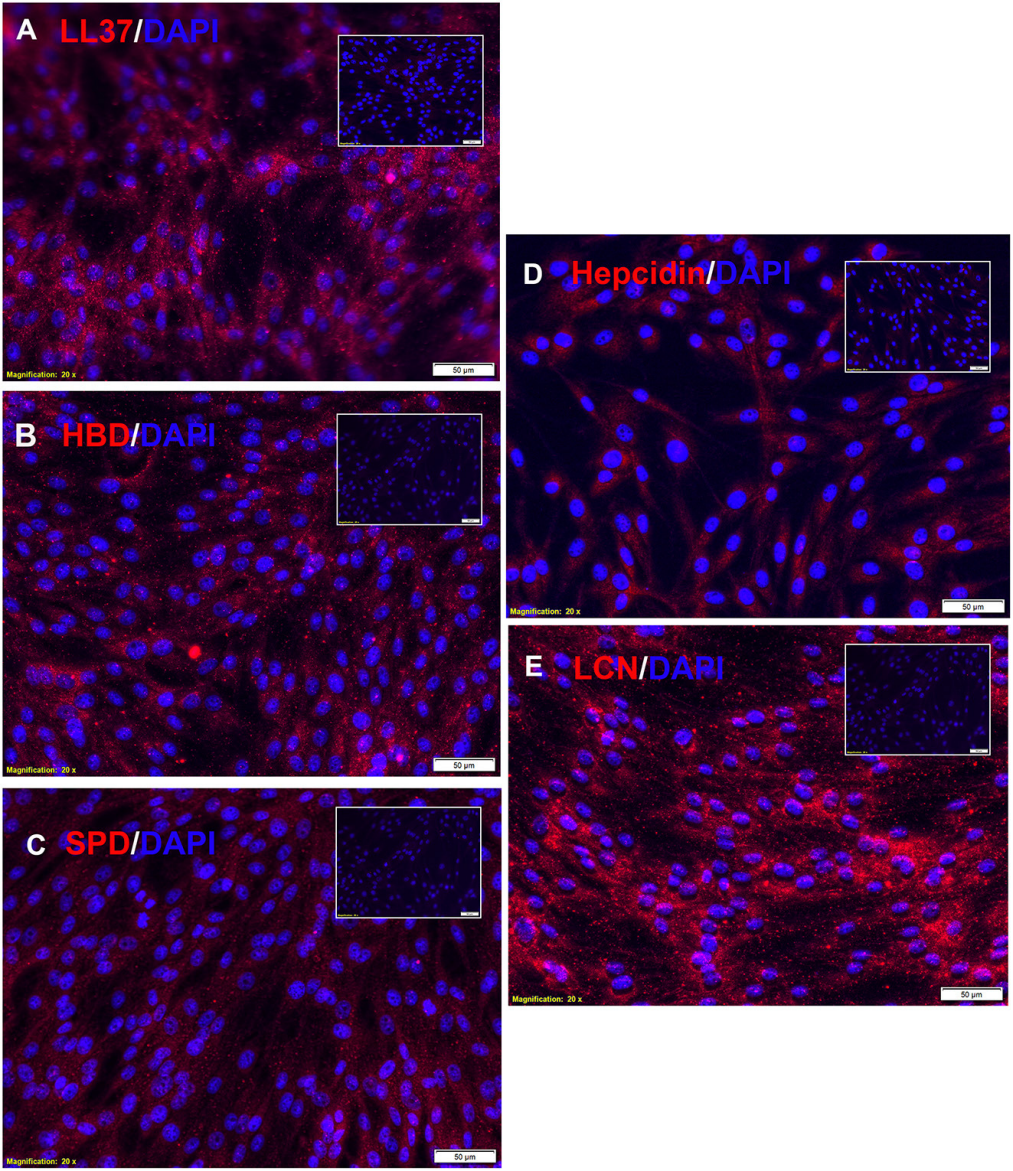
Intracellular expression of antimicrobial peptides by canine MSC. Canine adipose tissue derived MSCs were fixed and immunostained with antibodies to the 5 indicated antimicrobial peptides, as described in Methods. In each panel is depicted immunostaining with the antimicrobial peptide antibody (red), and a matched non-immune antibody. Cell nuclei are delineated by DAPI staining (blue). Scale bar and magnification listed on bottom of each panel. Expression of for **(A)** LL-37, **(B)** human beta-defensin, **(C)** surfactant protein D, **(D)** hepcidin, and **(E)** lipocalin.

Therefore, we investigated next the expression of antimicrobial peptides by canine MSC. Using immunohistochemistry (Figure 2A), we demonstrated the intracellular expression of LL-37. Canine MSC were also found to express high intracellular levels of lipocalin, an antimicrobial peptide commonly expressed by macrophages at mucosal surfaces (Figure 2E) (9). Lower levels of expression of beta defensin (Figure 2B), hepcidin (Figure 2D) and surfactant protein D (Figure 2C) were detected. Notably, activation of MSC with the TLR3 ligand pIC for 24 h did not increase intracellular expression of these 5 antimicrobial peptides above the levels expressed by non-activated MSC (data not shown), which is in contrast to findings from studies of human and equine MSC, where TLR activation has been shown to stimulate release of several antimicrobial peptides (7, 16).

### TLR Activation Stimulates MSC Migration and Cytokine Secretion

**Figure 3:**
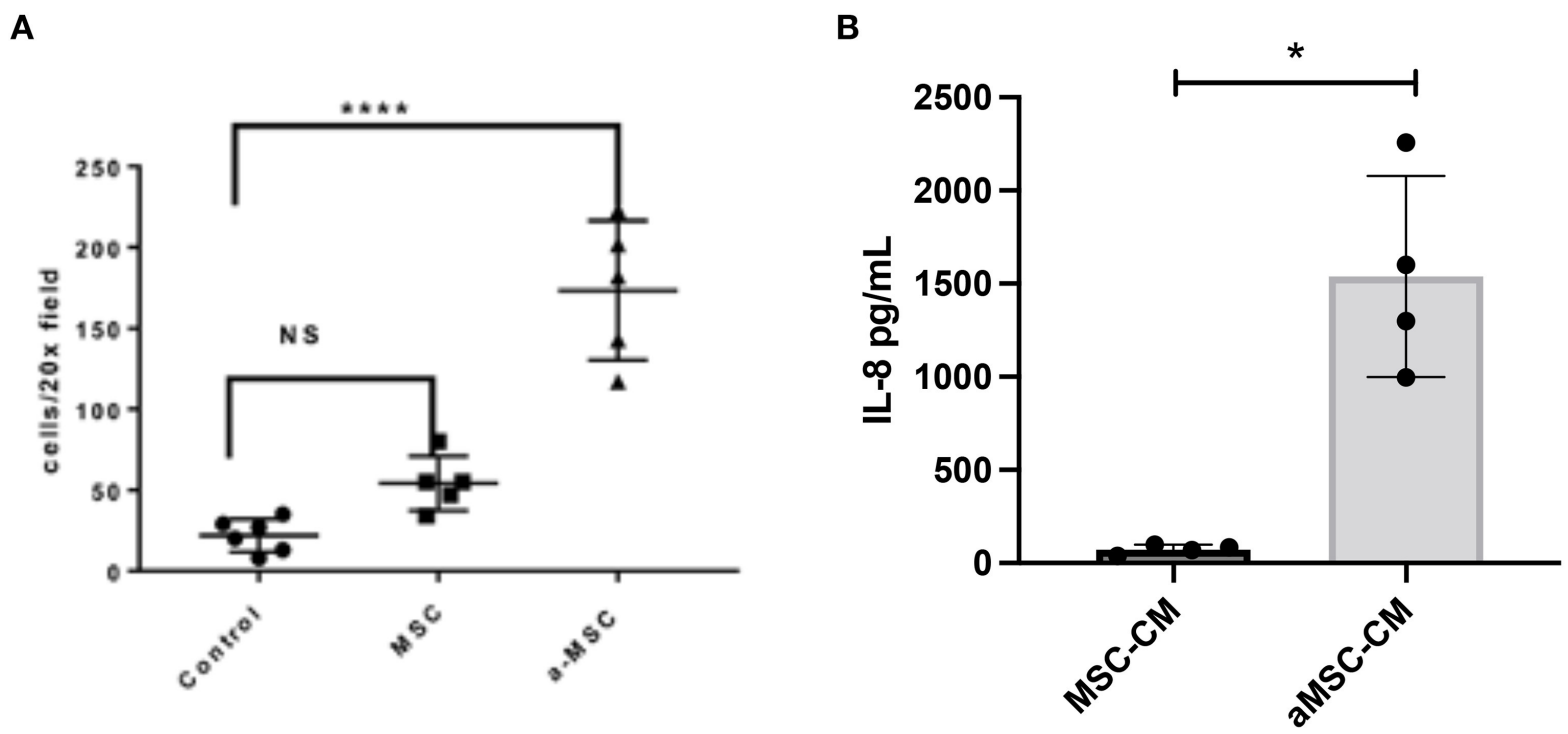
Impact of MSC activation on migration and cytokine secretion. Canine MSC were activated with the TLR3 ligand pIC, and the effects on migration and IL-8 secretion assessed, as detailed in Methods. In **(A)**, the average number of cells migrating towards an SDF-1 gradient as done in triplicate wells is shown. Non-activated and activated MSC were compared, as well as spontaneous migration across the transwell with no added SDF-1. Statistical comparisons for 3 groups were done using one-way ANOVA, followed by Tukey multiple means post-test, with statistical significance determined for ****P ≤ 0.0001. In **(B)**, IL-8 secretion by triplicate cultures of MSC was measured by specific ELISA, compared non-activated (white bar) and activated MSC (white bar) (checkered bar). *denotes *p* < 0.05 as determined by Mann-Whitney t test.

Previous studies by our group have shown that activating mouse adipose derived MSC increases MSC migration to sites of infection following i.v. delivery (10, 27). MSC migration to sites of inflammation appears to be driven by CXCR4 expression by MSC and secretion of the SDF-1 chemokine by inflamed cells (28). Therefore, we used *in vitro* migration assays to assess the impact of activation of canine MSC on their migratory activity (Figure 3). These studies revealed that MSC migration to an SDF-1 gradient was significantly increased when cells were activated with pIC (Figure 3A).

Secondly, we also investigated the impact of activation on the secretion of the key neutrophil chemokine IL-8 (CXCL8), which has been reported previously for human and equine MSC (7, 16). We found that activation significantly increased the secretion of IL-8 from MSC (Figure 3B). Taken together, these findings indicate that activation of MSC prior to their systemic administration can improve both their ability to migrate to sites of infection, and to release chemokines that recruit and activate host innate immune defenses.

### MSCs Secrete Factors That Induce Macrophage Polarization Toward an M2 Phenotype and Enhance Macrophage Bactericidal Activity

**Figure 4:**
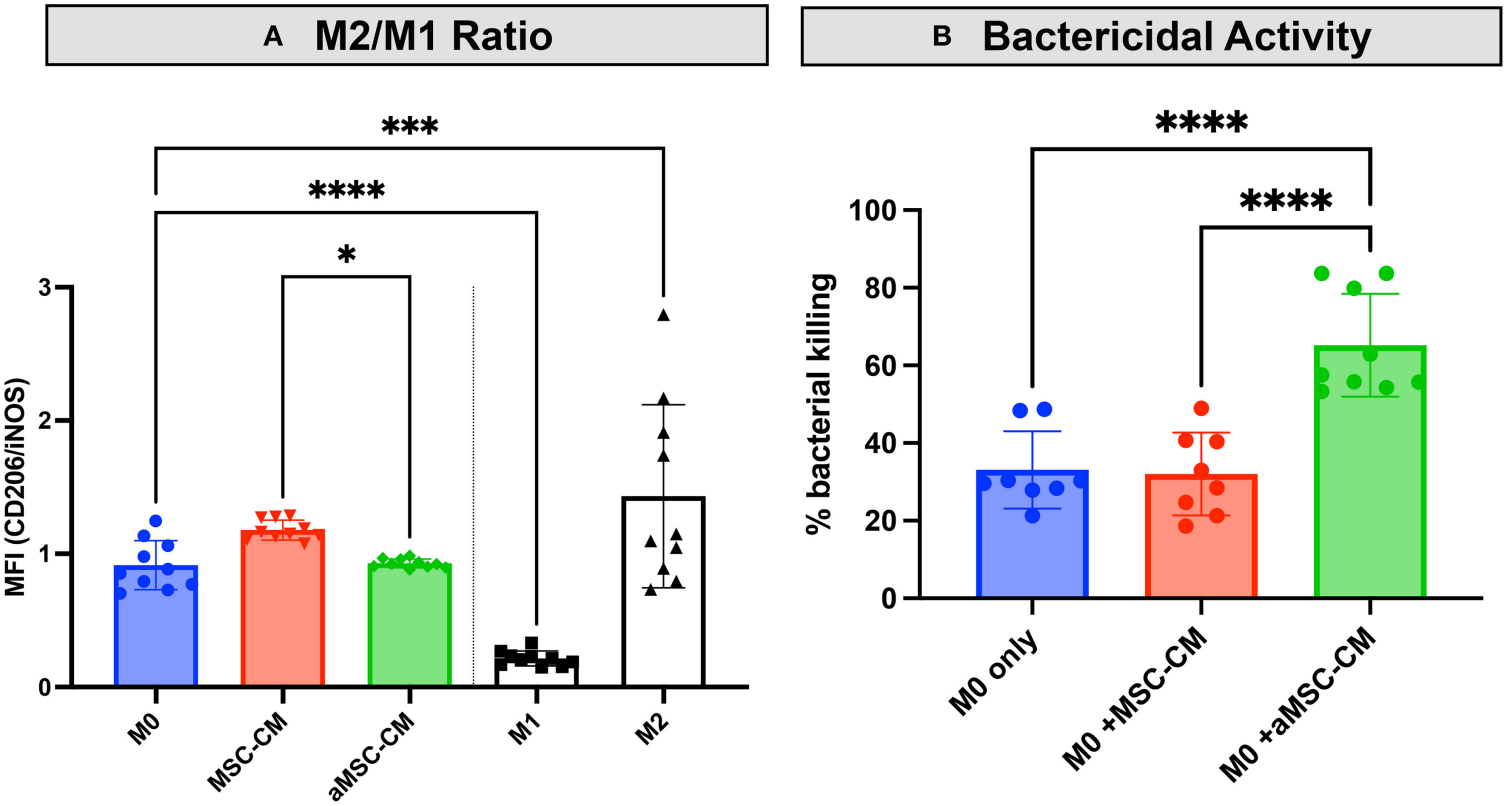
Impact of MSC CM from activated and non-activated MSC on macrophage bactericidal activity. Canine monocyte-derived macrophages were generated from healthy dogs as noted in Methods, and then incubated with CM obtained from 48 h cultures of activated (aMSC) and non-activated (MSC). In **(A)**, incubation with aMSC CM stimulated an M2 phenotype less effectively than MSC CM, which promoted M2 differentiation, compared to untreated macrophages. In **(B)**, the effects of MSC CM on macrophage bactericidal activity against *S aureus*, as described in Methods, were assessed, using macrophages incubated for 48 h with MSC CM. Incubation with aMSC CM was significantly more effective in stimulating macrophage bactericidal activity than MSC CM. Statistical comparisons were done using one-way ANOVA, followed by Tukey multiple means post-test, with statistical significance determined for *P ≤ 0.05, **P ≤ 0.01, ***P ≤ 0.001, ****P ≤ 0.0001.

MSC have been reported to alter the activation state of macrophages. Multiple groups report an increased M2 or wound-healing, anti-inflammatory type macrophage after interaction with MSC or MSC secreted factors (10, 29–31). To determine the effect of resting and activated MSC on canine macrophages, we incubated bone marrow derived macrophages with conditioned media from resting and activated MSC. Using expression of CD206 (macrophage scavenger receptor) and iNOS (inducible nitric oxide synthase enzyme) as markers of M2 and M1 macrophages respectively, we assessed the impact MSC CM on macrophage polarization. These studies revealed that MSC CM did indeed shift macrophages toward an M2 phenotype (Figure 4A), while aMSC CM treated macrophages compared to MSC CM more resembled M1 phenotype with a significantly lowered M2/M1 ratio compared to MSC CM. We also noted that macrophage incubation with MSC CM upregulated IL-10 production, but the amount produced did not differ between macrophages exposed to activated vs. non-activated MSC CM (data not shown).

Studies with human and rodent macrophages have demonstrated the stimulatory effects of MSC secreted factors on bactericidal activity (7, 18, 31, 32). To determine if MSC CM also altered canine macrophage bactericidal activity, canine macrophages were incubated with CM from resting or activated MSC for 48 h, and the effects on macrophage spontaneous bactericidal activity assessed, as described in Methods (23). These studies revealed that activated MSC CM significantly enhanced macrophage bactericidal activity, compared to incubation with resting MSC CM, or untreated macrophages (Figure 4B). Thus, this set of experiments revealed that activated MSC are able to significantly alter the polarization and functionality of macrophages, which is one of the possible mechanisms by which activated MSC may exert their activity against drug-resistant bacterial infections.

#### Secretion of Fibroblasts Stimulatory Factors by MSC

**Figure 5:**
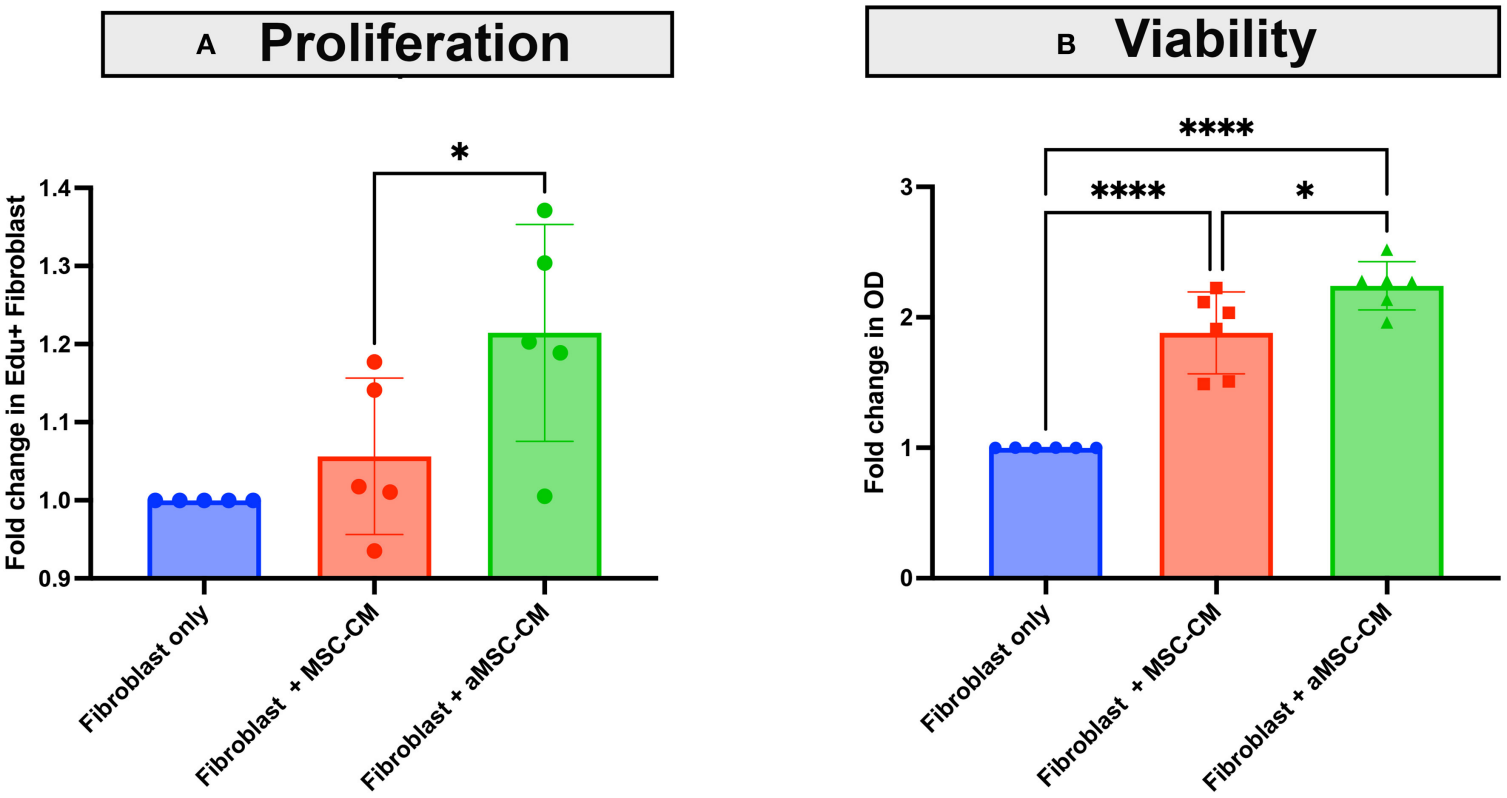
Effects of MSC CM on canine skin fibroblast proliferation and expansion. Skin fibroblasts cultures were established from skin biopsies from a normal dog, as described in Methods. In **(A)**, the impact of MSC CM on fibroblast proliferation was assessed over a 24 h period, using EDU incorporation and quintuplicate culture wells, as noted in Methods. Fibroblasts were incubated with MSC-CM (red) and aMSC-CM (green) or untreated cultures (blue), with dots representing the fold change in EDU incorporation by flow cytometry. In **(B)**, the impact of MSC CM on numbers of fibroblasts generated over a 24 h period, as assessed by MTT assay are depicted, with MSC-CM (red) and aMSC-CM (green) or untreated cultures (blue) shown. Each experiment was completed with 3 different canine skin fibroblast lines, and with MSC derived from 2 separate dogs. Significance was computed using 2-way ANOVA, with Tukey's multiple comparisons test to compare means within each time point. statistical significance was determined for *P ≤ 0.05.

Healing of an infected wound requires bactericidal activity to clear the infection, a macrophage influx to clear area of debris, and also re- epithelialization and granulation for full wound healing. Fibroblasts are an important component of wound healing through their role in generating granulation tissue, which involves fibroblast proliferation and local migration, and the beneficial effects of MSC or MSC CM on wound healing and fibroblast proliferation have been described in species other than dogs (33–36). Therefore, we examined the effect of MSC secreted factors on canine skin derived fibroblast proliferation and migration (Figure 5). Skin fibroblasts incubated with aMSC-CM, but not resting MSC-CM, significantly increased their proliferation rate as assessed by EDU incorporation (Figure 5A). The number of fibroblasts generated over a 24 h period also increased significantly following incubation with aMSC CM (Figure 5B).

### Impact of Repeated Systemic Administration of Activated MSC on Clinical and Microbiological Responses in Dogs With Chronic, Drug-Resistant Infections

**Figure 6:**
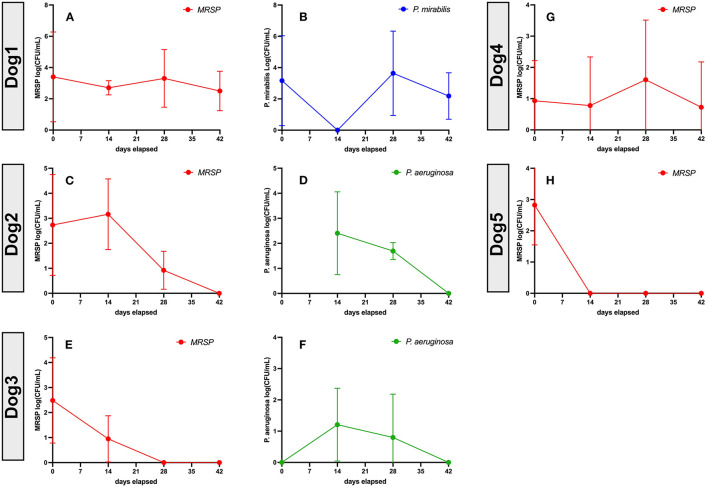
Bacteriologic response at 8 weeks in 5 dogs treated with activated MSC. Quantitative bacterial cultures were obtained from wounds from Dogs 1–5 (Table 1), as described in Methods. In each figure, the Y axis depicts the bacterial isolate and the average bacterial counts (CFU, with SD), while the X-axis depicts days in study and time points for sample collection. **(A,C,E,G,H)** show MRSP counts. **(B)** *P. mirabillis* counts in dog1. **(D,F)** *P. aeruginosa* counts. Dog 6 quantitative cultures were not performed as the joint was not amenable to 4 quadrant cultures, Dogs 7 and 8 were not included in this analysis because quantitative cultures could not be performed accurately from nasal swab samples. statistics were not used to make conclusions about this portion of the study as responder was defined as no culturable bacteria at conclusion of study.

To validate the earlier findings from a mouse chronic biofilm infection model that evaluated i.v. treatment with activated MSC (10), we conducted a pilot study of TLR3 activated MSC safety and efficacy in pet dogs with spontaneous, chronic (minimum 4 weeks duration), drug-resistant bacterial infections in a variety of different sites, including soft tissues and joints. Eight dogs were enrolled in the non-randomized, phase I/II trial, after meeting the enrollment criteria described in more details in Methods, including the presence for at least 4 weeks of antibiotic non-responsive, chronic infections, with documented multi-drug resistance. The clinical and microbiological results of the study are presented in Table 1 and Figure 6.

**Table 1:** Microbiological and clinical responses in 7 dogs with multi-drug resistant infections treated with activated MSC.

**Dog**	**Infection site**	**Infection duration prior to study entry**	**Conncurrent antibiotic**	**Bacterial species isolated**	**Clinical response at 8 weeks**	**Bacteriologic response at 8 weeks**
1	Post op limb spare surgical site dehiscence	1 year	Cephalexin	MRSP PR	**Responder**−50% wound contraction	**Non-responder**–Proteus and MRSP decreased by 10%
2	Post op wound repair dehiscence	6 weeks	Marbofloxacin, Chloramphenicol	MRSP PA	**Responder**−75% wound contraction	**Responder**–eliminated infection
3	Post op wound repair dehiscence	8 weeks	Amoxicillin/ clavulanic acid, Marbofloxacin	MRSP PA EC	**Responder**–fully healed	**Responder**–eliminated infection
4	Decubital ulcer	4 weeks	NA	MRSP	**Responder**−80% contraction of wound	**Non-responder**–no change in quantitative count
5	Wound antebrachium	8 weeks	Chloramphenicol, Enrofloxacin	MRSP	**Responder**–fully healed	**Responder**–eliminated infection
6	Infection in tibiotarsal joint	6 months	Clindamycin	MRSP	**Responder**–joint cytology normal at 8 weeks	**Responder**–eliminated infection
7	Nasal infection following radiation therapy	1 year	Marbofloxacin	MRSP EC, Entc, Strep	**Non-responder**−10% improvement in nasal score	**Partial responder**–eliminated all bacteria but MRSP
8	Nasal infection following radiation therapy	1 year	Trimethoprim Sulfamethoxazole	MRSP, EC, Entc, Kleb pn, Crn	**Responder**−50% improvement in nasal score	**Partial responder**–**e**liminated all bacteria except MRSP and EC

*Organisms isolated from the wounds are abbreviated as follows: MRSP, methicillin resistant Staphylococcus pseudointermedius; EC, Escherichia coli; PA, Pseudomonas aeruginosa; Crn, Corynebacterium species; PR, Proteus species; Ent, Enterobacter species; Entc, Enterococcus species; Strep, Streptococcus species; Kleb pn, Klebsiella pneumoniae*.

**Figure 7:**
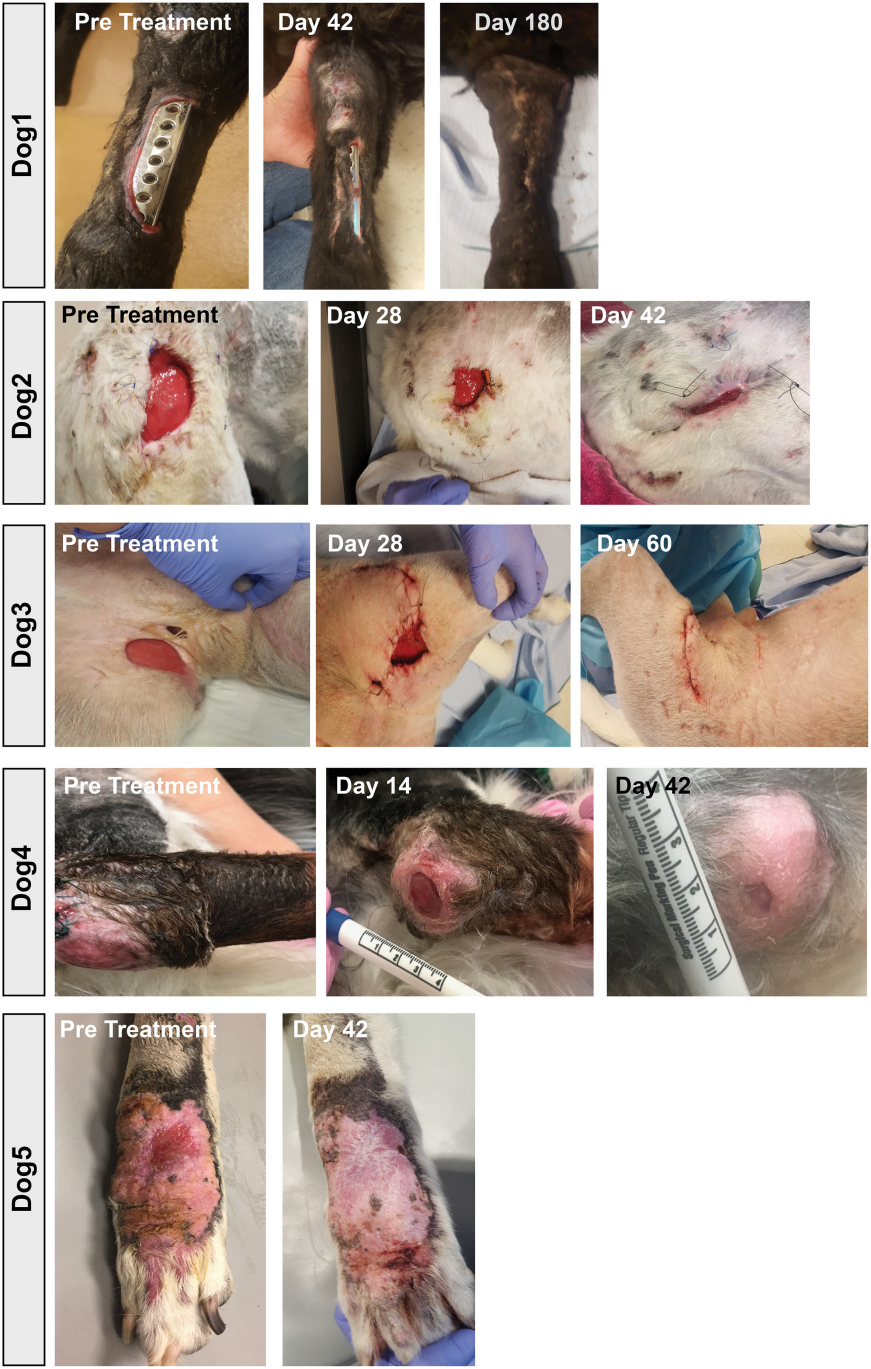
Wound healing in dogs with chronic infections treated with activated MSC. Serial photographs of drug-resistant infections in 5 of the 7 dogs in the study, treated with activated MSC. Two dogs with nasal infections were not included as the infection was confined to the internal nares and could therefore not be photographed.

Among the key findings from this study were first that repeated (at least 3 times) i.v. infusions of activated, allogeneic MSC were very well-tolerated, with no adverse events reported for any of the 8 study dogs. In addition, 7 of 8 treated dogs experienced clinical benefit (wound healing, infection site clearance, return of function, decreased odor and amount of discharge in nasal infection) over the course of the 8-week study (Table 1). Wound healing typically required at least 20–40 days from the first MSC treatment for most treated animals, depending on the size of the initial infected site or wound (Figure 7). In some cases, additional MSC treatments beyond the initial 3 were required, as was the case for Dog 1 (Figure 7), which received a total of 7 treatments over 9-months before the wound fully healed over the exposed bone plate.

Also notable was the microbiological improvement (partial or complete bacterial clearance) experienced by 4 of the 8 treated dogs (Table 1 and Figure 6), comparing pre MSC treatment to the end of the 8 week trial period. For 3 of these dogs, quantitative cultures show complete elimination by 42 days post treatment (Figures 6C–F,H). This response rate is similar to what was reported previously in an earlier trial with 7 dogs with spontaneous drug resistant infection, in which 5 of 7 of those treated dogs also cleared their infections (10). Even in animals where full microbiological clearance was not achieved (e.g., Dog 1 and Dog 4), there was still clinical improvement in wound healing (wound contraction, decrease in wound diameter Figure 7), suggesting that the wound healing effects of activated MSC treatment may not only result from bacterial clearance, but likely involve other pathways as reported previously (34, 36).

## Discussion

A number of prior publications have described the anti-infective, immunomodulatory and wound healing properties of MSC, in multiple different species (6, 7, 9–12, 14, 15, 17, 18, 30, 37, 38). However, the current study is the first to our knowledge to examine the anti-microbial properties of canine adipose derived MSC in detail. These studies therefore included evaluation of the direct secretion of antimicrobial factors and generation of bactericidal activity by canine MSC, as well as the interaction of MSC secreted factors with conventional antibiotics for eliciting enhanced bactericidal activity. In addition, this study evaluated the indirect effects of MSC factors on macrophage functional and bacteriological properties, and finally the effectiveness of repeated systemic therapy with activated canine MSC for treatment of drug-resistant infections in a clinical study in pet dogs with spontaneous infections. Key findings include demonstration of direct MSC bactericidal activity by canine MSC, as well as activation of macrophage bactericidal activity and fibroblast proliferation by MSC, and to a greater degree by activated MSC, and secretion of key antimicrobial cytokines including IL-8.

We previously reported in a mouse *S. aureus* biofilm infection model that treatment with activated MSC, when combined with antibiotics (amoxi-clav), could clear the infection and stimulate wound healing, whereas treatment with non-activated MSC had significantly less activity (10). Therefore, in the present study with dog MSC, we evaluated many of these same antimicrobial properties. We observed, that compared to murine or human or equine MSC, canine MSC overall generated substantially less direct bactericidal activity (7, 10, 16). This effect likely results from reduced secretion of antimicrobial peptides (AMPs) such as LL-37, though LL-37 secretion was not compared directly across species. Another important difference between dog MSC and mouse and human MSC is that TLR3 activation of canine MSC had less impact on generation of direct antimicrobial activity, though it is important to note that TLR3 activation did significantly enhance the indirect antimicrobial properties of canine MSC. Although the levels of AMPs tested (LL37, HBD, SPD, Hapcidin, LCN) were not increased with TLR3 activation, a recent study examining antimicrobial properties of canine bone marrow derived MSCs (39) showed other classes of antimicrobial peptides such as apolipoprotein B and D, amyloid-β peptide, and S100-A4…etc. All of which have the potential to contribute to the direct antimicrobial activity; however full proteomic sequencing may be needed to determine the full continuum of peptides screted following TLR3 activation. AMPs have also been demonstrated to work synergistically with common antimicrobial agents (40). It is likely that production of these AMPs is responsible for the observed synergistic effect *in vitro* with MSC and antibiotics but further testing would be necessary to demonstrate this association.

Neutrophils play a large role in infection, and previous studies in mice have demonstrated increased neutrophil phagocytosis, increased longevity and increased production of oxygen free radicals in neutrophils when the neutrophils were exposed to conditioned media from activated stem cells (41, 42). We discovered an increased secretion of IL-8, a chemokine that promotes recruitment of neutrophils in TLR3 activated MSC. Increased neutrophil migration together with enhanced bactericidal function likely plays a role in the demonstrated bactericidal activity.

When canine macrophages were exposed to MSC conditioned media we observed a trend toward increased M2 macrophages wheras activated conditioned media resulted in a slightly more M1 phenotype. However, these same macrophages when exposed to aMSC-CM did not secrete TNFα which is generally associated with an M1 or inflammatory macrophage. Although macrophages are classified *in vitro* utilizing surface markers generally associated with inflammatory and anti-inflammatory macrophages, we know this process to be a continuum and as such macrophages *in vivo* are likely in a state of flux and not entirely inflammatory or anti-inflammatory (43). Indeed, some studies have demonstrated that exposure of macrophages to MSC induce an alternative state that has properties of both types of macrophages although it is skewed toward M2 (44). Further supporting a wound healing role of MSC were the response of canine fibroblasts to aMSC secreted factors. These somewhat paradoxical results demonstrate that *in vitro* aMSC have effects on macrophages that may have elements of both inflammatory (M1) and anti-inflammatory (M2) macrophages.

In addition to the indirect antimicrobial properties, MSC activation also increased their migration to an inflammatory stimulus (i.e., SDF-1 gradient; Figure 3). Increased stem cell migration toward the site of inflammation and infection would increase numbers of stem cells at that site. Thus, while canine MSC resembled those of other species with respect to their direct and indirect antimicrobial activity, there were also important species-specific differences.

The clinical study in dogs with spontaneous drug-resistant bacterial infections, though only a relatively small number of animals were evaluated, confirmed and extended results from a previous study of activated MSC therapy in dogs (10). It is likely that the observed clinical response of resolution or decrease of infection with healing of infected wounds represents a result of a combination of factors likely involving increased antibacterial activity from antimicrobial peptides, amplified macrophage killing and phagocytosis, and accelerated neutrophil migration and killing, especially considering the I.V administration of MSC and previously reported paracrine effects on would healing (45). *In vivo* studies in large animals are limited, however various *in vitro* study data across species confirms the multifactorial effects of MSCs (17). Limitations to the current study included the small number of animals evaluated (*n* = 8), as well as the non-randomized recruitment of study animals, which could have introduced a number of potential study biases. However, we did attempt to address the issue of study confounding by changes in antibiotic treatment protocols during the study by eliminating the option to change antibiotics (except in the case of progressive infection) during the 8-week study period, thus keeping the antibiotic treatment protocol constant throughout. A larger study, with random allocation of dogs to study groups, would be required to address the bias issues, though such as study may be difficult to design due to need for fixed antibiotic protocols for the untreated control group.

These study findings have important implications not only for clinical management of drug-resistant infections in dogs, but also for treatment of drug-resistant infections in humans, which have been steadily increasing in frequency and severity (2, 3). Given that human MSC appear to generate higher direct antimicrobial activity, and respond better to activation, it may be plausible to consider the effectiveness of the activated MSC treatment strategy for difficult to treat chronic, drug resistant infections in humans, combined with antibiotic therapy. Several clinical trials have reported previous or ongoing work to evaluate MSC for treatment of acute infections, including bacterial pneumonias (14, 46). Therefore, it would be informative to consider similar studies of activated allogeneic MSC therapy for recalcitrant infections, including soft tissue infections and deep bone infections, in humans.

Taken together, the findings reported here extend those reported previously for dogs and mouse models and provide greater insights into several different potential mechanisms of action for activated MSC in chronic infections with biofilm formation. Indeed, it is likely that multiple complementary mechanisms account for the overall activity of MSC therapy for treatment of these complex, drug-resistant infections, including both direct (locally acting antimicrobial peptides) and indirect (systemic activation of host innate immune effector cells) effects on local and distant immune and antimicrobial mechanisms.

## Data Availability Statement

The original contributions presented in the study are included in the article/supplementary material, further inquiries can be directed to the corresponding author.

## Ethics Statement

The animal study was reviewed and approved by CSU Institutional Animal Care and Use Committee. Written informed consent was obtained from the owners for the participation of their animals in this study.

## Author Contributions

VJ and JH: animal exams. LC, SD, and VJ: manuscript writing. JH, SD, and VJ: collection and assembly of data. SS and LC: collection of data. SD and VJ: conception, design, and provision of study material or patients. SS, LC, SD, and VJ: data analysis and interpretation. SD: final approval of manuscript. All authors contributed to the article and approved the submitted version.

## Conflict of Interest

The authors declare that the research was conducted in the absence of any commercial or financial relationships that could be construed as a potential conflict of interest.

## Publisher's Note

All claims expressed in this article are solely those of the authors and do not necessarily represent those of their affiliated organizations, or those of the publisher, the editors and the reviewers. Any product that may be evaluated in this article, or claim that may be made by its manufacturer, is not guaranteed or endorsed by the publisher.
